# Towards Sustainable Protein Sources: The Thermal and Rheological Properties of Alternative Proteins

**DOI:** 10.3390/foods13030448

**Published:** 2024-01-31

**Authors:** Kaitlyn Burghardt, Tierney Craven, Nabil A. Sardar, Joshua M. Pearce

**Affiliations:** 1 Department of Chemical & Biochemical Engineering, Western University, London, ON N6A 5B9, Canada; 2 BeeHex, LLC, Columbus, OH 43230, USA; 3 Department of Electrical & Computer Engineering and Ivey Business School, Western University, London, ON N6A 5B9, Canada

**Keywords:** rice protein, spirulina protein, pea protein, soy protein, plant-based diet, thermal properties, rheological properties, protein processing

## Abstract

Reducing meat consumption reduces carbon emissions and other environmental harms. Unfortunately, commercial plant-based meat substitutes have not seen widespread adoption. In order to enable more flexible processing methods, this paper analyzes the characteristics of commercially available spirulina, soy, pea, and brown rice protein isolates to provide data for nonmeat protein processing that can lead to cost reductions. The thermal and rheological properties, as well as viscosity, density, and particle size distribution, were analyzed for further study into alternative protein-based food processing. The differential scanning calorimetry analysis produced dry amorphous-shaped curves and paste curves with a more distinct endothermic peak. The extracted linear temperature ranges for processing within food production were 70–90 °C for spirulina, 87–116 °C for soy protein, 67–77 °C for pea protein, and 87–97 °C for brown rice protein. The viscosity analysis determined that each protein material was shear-thinning and that viscosity increased with decreased water concentration, with rice being an exception to the latter trend. The obtained viscosity range for spirulina was 15,100–78,000 cP, 3200–80,000 cP for soy protein, 1400–32,700 cP for pea protein, and 600–3500 cP for brown rice protein. The results indicate that extrusion is a viable method for the further processing of protein isolates, as this technique has a large temperature operating range and variable screw speed. The data provided here can be used to make single or multi-component protein substitutes.

## 1. Introduction

Although the UN reports that the annual population growth rate has been dropping (and is expected to keep dropping), the total population of the planet is expected to surpass 10 billion people in the 2050s [1]. This increasing population prompts the need for more sustainable sources of food [2] and, perhaps, the most challenging need: a low-cost source of abundant protein [3]. It is now widely accepted that meat production uses more energy and has a greater negative environmental impact than plant-based meat alternatives [4]. The average American meat-based diet demands more energy, land, and water resources in comparison to an ovo-lacto vegetarian diet, which underscores the need to shift from current meat-based food systems to sustainably meet the dietary demands of the increasing global population [5]. In addition to poor protein conversion efficiency, meat-based diets are unhealthy [6,7,8], whereas vegetarian diets are associated with a long list of health benefits, including lower rates of death from ischemic heart disease, lower cholesterol levels, lower blood pressure, and lower rates of hypertension and type 2 diabetes [9]. There are also public concerns about animal welfare [10,11] and serious public health issues related to animal diseases and animal husbandry practices that result in antibiotic-resistant bacteria [12,13]. Finally, agriculture is a major contributor to greenhouse gas (GHG) emissions; specifically, methane accounted for 35% of total food system GHG emissions in 2015 [14]. Livestock is a leading source of global methane emissions, and in 2010, 23% of global temperature warming was attributed to livestock emissions [15].

For these reasons, a more efficient plant-based protein diet may be used to mitigate agriculture’s contribution to GHG emissions and other environmental hazards [16,17,18]. This relates to UN Sustainable Development Goal 1, as the cost of animal-based protein has increased globally but can be particularly inaccessible because of costs in the developing world. Consumers have been shifting towards this as the plant-based market [19] has grown from USD 4.8 billion in 2018 to USD 7.4 billion in 2020 [20]. Although clinically vegan/vegetarian diets have proven healthy for humans [21] and the planet [22], converting to a plant-based diet is challenging for some [23,24]. In order to overcome this challenge, a range of plant-based meat substitutes is under development [25,26,27] and have been commercialized (Beyond Meat, Impossible Burger, etc.) [28]. Unfortunately, these meat substitutes are often more expensive than the meat they aim to replace. For example, at Walmart Canada, 340 g of Beyond Meat Ground Beef is CAD 7.97 (2.3 cents/g), while multiple 450 g farm ground beef options range from CAD 4.97–7.47 (1.1 cent/g to 1.6 cents/g), which substantially restricts their uptake [29,30]. Yet, alternative protein on the market are generally substantially less costly than meat protein, as shown in Table 1. The protein cost was calculated by dividing the purchase cost by the protein content. Note that the purchase cost has been converted from the reported USD/lb to USD/kg.

As can be seen in Table 1, the wholesale price of plant protein ranges from 0.00294 USD/g protein (rice)–0.0142 USD/g protein (spirulina), while meat purchase cost has an overall more expensive range of 0.0203 USD/g protein (chicken)–0.0946 USD/g protein (lamb) (Table 1). The most inexpensive plant protein cost is derived from the protein concentration of the raw source material without considering any protein isolation method costs. Raw green peas are the exception to this trend due to their low protein content in comparison to other plant sources. Overall, soy is the most inexpensive plant protein, followed by pea, rice, and spirulina, which are the most expensive. In order to make these meat alternatives more appealing, they normally undergo substantial processing. In order to make meat substitutes more accessible, the basic thermal and rheological properties are needed for alternative sources of protein and their processing, such as extrusion [58]. This paper analyzes the characteristics of spirulina, soy, pea, and brown rice proteins; these compounds naturally grow in various parts of the world and are commercially available. The thermal and rheological properties, viscosity, density, and particle size distribution are analyzed for feasibility comparisons that can be used for further study on alternative protein-based food processing and production. This study is important, as it analyzes more sustainable sources of alternative protein, which can be used to reduce the environmental footprint of the agriculture industry. Specifically, this work provides a means of using economic screw extruders to process a wide range of plant-based proteins into low-cost edible food.

## 2. Materials and Methods

### 2.1. Materials

The alternative protein materials were sourced from Healthy Planet Canada and include spirulina [42], soy protein isolate [47], pea protein isolate [52], and rice protein isolate [57].

### 2.2. Density

The densities of all the materials were determined to provide a full characterization by (1) massing an empty one dram (3.69669 cm^3^) vial with a digital scale, (2) filling the vial with the material, and packing it down to flatten the top and record the mass; (3) the bulk density (d) was given by the equation below, where m is mass and V is volume:

(1) $$d[\frac{g}{{cm}^{3}}]=\frac{m_{full\;vial}-m_{empty\;vial}}{V_{vial}}$$

### 2.3. Particle Size

The particle size was quantified to provide the limits for material processing when using the materials (e.g., 3D printing resolution is limited by the maximum particle size). By using a digital microscope (Celestron, Torrance, CA, USA), a micrometer calibration slide (Walfront, Lewes, DE, USA) was imaged and imported into the open source ImageJ package (version 1.53) [59], and a line was drawn on the length of one division to provide the scale for all photos (938 pixels/mm). All five materials were then imaged three times using the same magnification of 200×.

It should be noted that when placing the protein particles on the microscope slides, they often clumped together. To ensure even distribution, after the particles were placed onto the slide, they were brushed off slightly. The images and the scale were imported into ImageJ and analyzed. Firstly, the color threshold of the particles was adjusted to differentiate them from the background. Depending on the image, a threshold method was chosen. The Shanbhag method [60] was the most accurate, which selected only the particles and no additional area in the background. For some images, the Shanbhag method did not work, so other methods, such as the Intermodes method [61], were used. The particle size distribution was determined for each protein sample, and the mean particle size was summarized.

### 2.4. Differential Scanning Calorimetry (DSC) Analysis

Differential scanning calorimetry was conducted using a Mettler Toledo (Columbus, OH, USA) DSC 3 and the DSC STARe System to analyze the thermal stability of the materials by obtaining endothermic peak temperatures. The DSC samples were both wet and dry. The dry samples were unchanged from the retail purchase. The wet (paste) samples were prepared by measuring 100–150 mg of each material into a clean aluminum boat and adding 50% by weight deionized water with a clean glass pipette. The paste was thoroughly mixed for 1 min. The samples were prepared freshly immediately before each paste DSC run. A total of 1.56–4.64 mg of each powder sample and 3.98–6.38 mg of each paste sample were weighed accurately and placed into an aluminum DSC pan and hermetically sealed. An empty pan was also hermetically sealed to be used as a reference. Both pans were placed into the module for the experiment. All samples were heated from 20–150 °C at a heating rate 5 °C/min. Nitrogen was used as a purge gas at flow rates of 30 or 50 mL/min, depending on the run, to clear out the materials for the next run. All DSC runs were analyzed to find the denaturation temperature (endothermic peak temperature) and the stable temperature processing ranges.

### 2.5. Viscosity Measurements

A Brookfield (MA, USA) DV-II+ with an S62 spindle viscometer was used to test the materials. A total of 600 g of the sample was measured and mixed with water to reach the desired concentration, referred to as Composition A. This sample slurry was transferred to a 600 mL glass beaker to undergo testing with the viscometer. The spindle was set 1.1 inches above the bottom of the beaker. The viscometer was set at RPM values of 5, 15, 25, 40, and 80. After testing concluded for composition A, additional water was added to reach composition B in the interest of saving sample material. The same experiment was duplicated with the new composition B. This method was repeated for all samples. A summary of compositions tested is shown in Table 2.

## 3. Results

Each of the material tests for each of the alternative proteins was provided separately below. It should be pointed out that the results provide the processing windows and types for each of the materials, and these are evaluated independently. The primary purpose of this study is to identify the processing windows (e.g., temperature and viscosities) that can be used to reduce the costs of alternative proteins. The following properties are quantified: density, particle size, DSC, and viscosity.

### 3.1. Density

The density of the alternative proteins is shown in Table 3.

### 3.2. Particle Size

**Figure 1:**
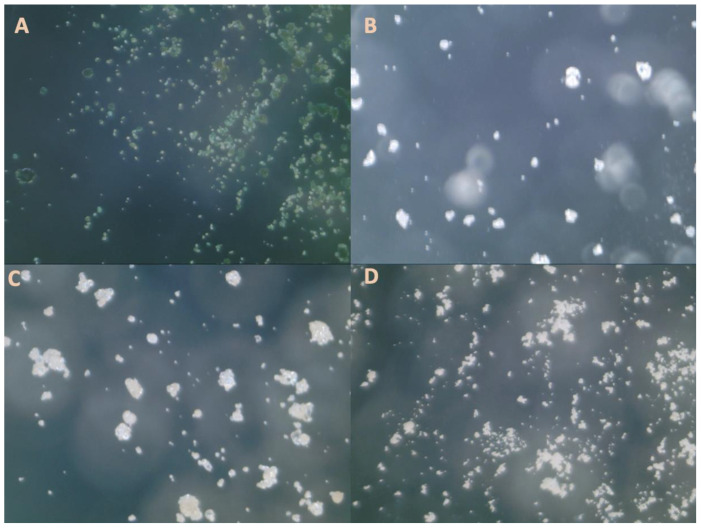
Digital microscope image with a magnification of 200× for (**A**) spirulina, (**B**) soy protein, (**C**) pea protein, and (**D**) brown rice protein.

**Figure 2:**
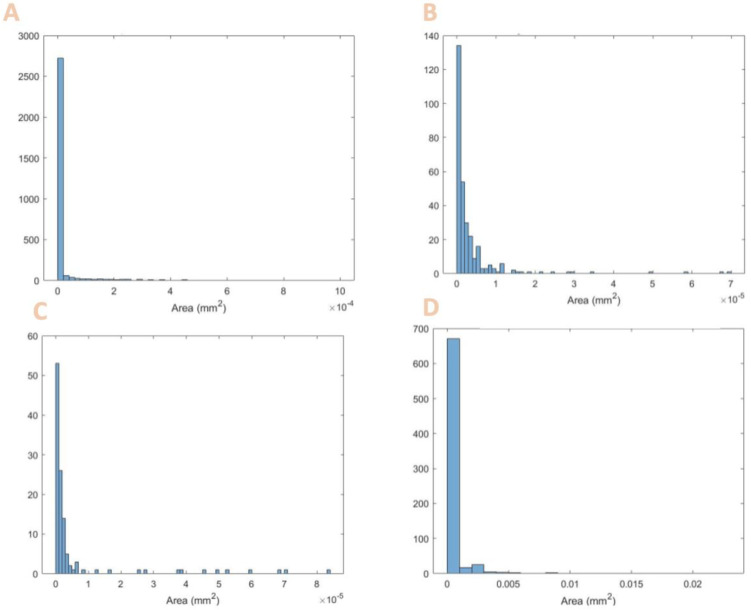
Histograms of particle size distribution for (**A**) spirulina, (**B**) soy protein, (**C**) pea protein, and (**D**) brown rice protein.

Figure 1 shows the micrographs, and Figure 2 shows the histogram of particle size distribution for each protein powder.

Table 4 summarizes the particle size and provides the literature values for comparison.

As seen from Table 4, the diameters found fit the range of known values for that particle. Although some are the minimum accepted values, they still fit within the range. The smaller particle sizes do enable some advantages in processing higher-resolution features.

### 3.3. DSC

**Figure 3:**
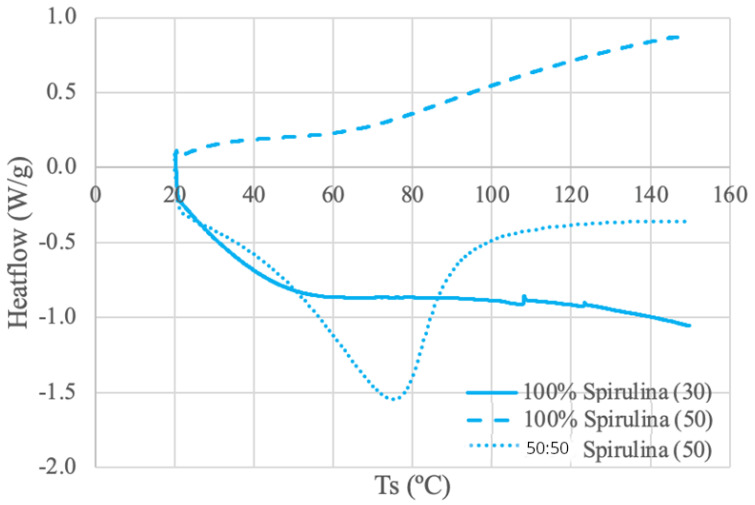
Spirulina DSC curves. The values (30) and (50) denote the nitrogen flow rate in mL/min.

**Figure 4:**
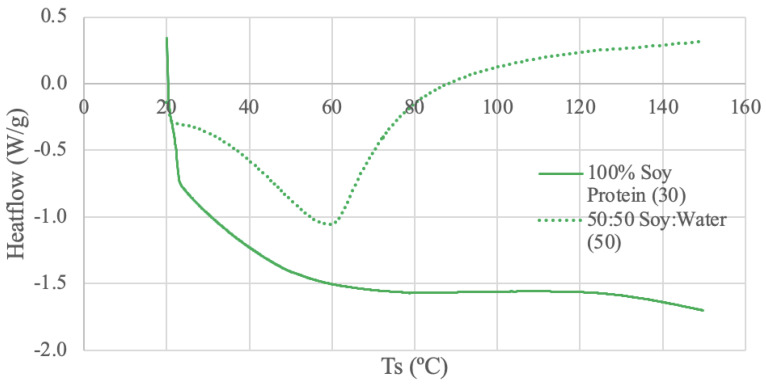
Soy Protein DSC curves. The values (30) and (50) denote the nitrogen flow rate in mL/min.

**Figure 5:**
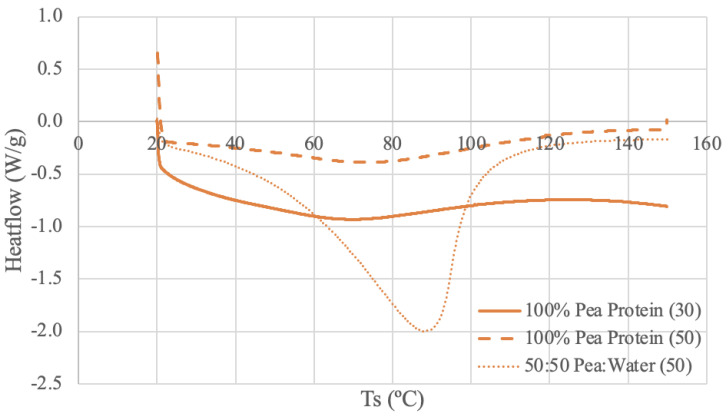
Pea Protein DSC Curves. The values (30) and (50) denote the nitrogen flow rate in mL/min.

**Figure 6:**
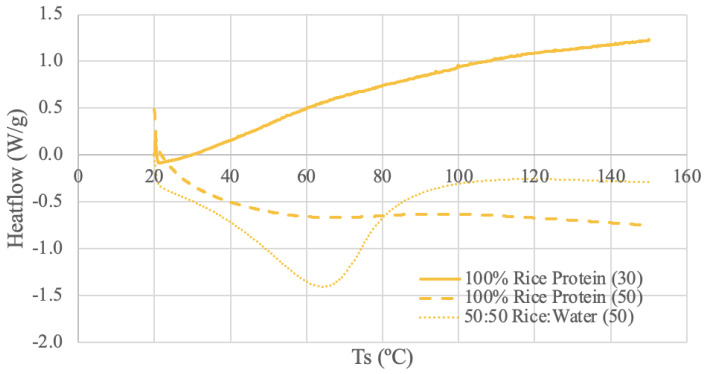
Brown Rice Protein DSC Curves. The values (30) and (50) denote the nitrogen flow rate in mL/min.

The DSC results for each protein source are shown in Figure 3, Figure 4, Figure 5 and Figure 6.

#### 3.3.1. Spirulina DSC Results

The spirulina protein samples were run under three different conditions: A 100% spirulina powder using 30 mL/min N_2_ and 50 mL/min N_2_, and a 50–50 ratio of spirulina and deionized water paste using 50 mL/min N_2_. Despite having an overall different curve shape, both non-50:50 powder samples exhibit a relatively linear trend line between 70–90 °C. Thermal deviations are evident in the spirulina (30) curve at around 107 °C and again at around 125 °C. This activity may be interpreted as possible thermal transition temperatures. Alternatively, the slight spikes may be an electrical effect and a result of static electricity discharge, although no significant power disturbance was observed [66]. A DSC study using 4 mg of spirulina protein isolated from spray-dried algal powder and dissolved in a 20 mL buffer solution displayed a DSC curve with a similar stability range of 73–83 °C [67]. The same curve also illustrated an endothermic melting phase change at 108.7 °C, like the spirulina (30) [67]. The DSC analysis in this study used a heating rate of 10 °C/min over a temperature range of 30–180 °C [67]. There are noticeable differences between the literature and obtained results, which can most likely be attributed to the differences in sample preparation and source material. Moreover, the different shapes of the powder curves may be attributed to increased heat transfer through an increased purge gas flow rate [68].

The spirulina and water paste curve looks like a more typical DSC curve with an obvious baseline and clear endothermic peak around 76 °C. In comparison, the dry samples lack the obvious thermal deviation present in the paste, demonstrating the amorphous shape of the dry curves [66]. Further, the difference in curve shape between the paste and powder samples suggests that a lower water content results in a lower denaturation enthalpy (∆Hd), assuming the peak area is equal to the enthalpy of denaturation [69]. This same result was reflected in a study using water and soybean protein [70]. Increasing heat promotes denaturation, and proteins unfold and lose their structure during denaturation [70]. Dehydration was determined to increase destabilization in the unfolded state [70]. Compared to the folded state, the unfolded state permits more contact between the compound and water [70]. Therefore, the unfolded state should have more internal bonding and be more compact at low water levels, which results in a lower ∆Hd [70]. Moreover, the paste curve’s endothermic peak (76 °C) is located within the assumed stable temperature range of the dry samples (70–90 °C). This discrepancy may be attributed to the decrease in water content. The lack of water may result in a rise in denaturation temperature (Td), which is also due to the destabilization of the unfolded states [70].

#### 3.3.2. Soy Protein DSC Results

The soy protein samples were run under two different conditions: dry powder using 30 mL/min N_2_ and a 50–50 ratio of soy protein and deionized water using 50 mL/min N_2_. The dry sample thermogram does not exhibit distinct peaks or deviations, but a plateaued temperature range is exhibited from 87–116 °C. The dry sample curve also has a large endothermic start-up hook, which may be attributed to calibration between the sample and reference pans [71]. This large start-up sloping baseline can hinder the detection of weak transitions [71]. The DSC study using soybean protein extracted from soybean flour exhibits similar-looking curves, as obtained for low water contents (1% and 5% water content) [70]. Little thermal activity was observed for the soy globular protein samples at low water contents, and only after 150 °C were any deviations visible, which is outside the temperature range of Figure 4 [70]. Another soybean protein DSC study using silver pans and a heating rate of 5 °C/min from 25 to 200 °C also found similar results [72]. Soybean protein isolates with low water content (11%) did not exhibit an endothermic peak until past 180 °C [72].

The half soy protein and half deionized water sample exhibit an endothermic peak at 59 °C. The thermal activity of the paste sample is more distinct than that of the dry sample, likely due to the increased water content [70]. Further, the slope of the curve increases after the peak, which suggests a shifted baseline [71]. Baseline shifts can be caused by changes in specific heat, which is evidence that the sample has gone through a transition, such as melting [71]. A different DSC study using soybean protein extracted from soybean flour and mixed with distilled water at a 50–50 ratio demonstrated an endothermic peak of approximately 110 °C [73]. The DSC conditions for this study were a heating rate of 10 °C/min between 20 to 130 °C, a nitrogen flow rate of 50 mL/min, and aluminum pans [73]. Despite the similar water content and DSC conditions, the endothermic peak temperatures are not similar, which can likely be attributed to the differences in sample source and preparation. In the literature, the water-protein paste was stored for 24 h at 4 °C in polyethylene bags to promote the even distribution of water, rather than just mixing followed by testing [73]. Another DSC study comparing corn starch and soy protein ratios with 80% water content and a heating rate of 5 °C/min from 25 to 150 °C also found different results [74]. Despite having a water content of 80%, no endothermic peak was observed for the pure soy protein and water sample, possibly due to the previous heat treatment of the soy in the manufacturing stages [74].

#### 3.3.3. Pea DSC Results

The pea protein samples were run under three different conditions: pure powder using 30 mL/min N_2_ and 50 mL/min N_2_ and a 50–50 ratio of pea protein and deionized water paste using 50 mL/min N_2_. Both dry samples have similar curve shapes and an indication of thermal activity through a broad endothermic peak from 67–77 °C. Pea protein (50) has a longer endothermic start-up hook, potentially due to a larger baseline adjustment requirement because of the increased purge flow rate [71]. The difference in heat flow levels is likely attributed to the varying nitrogen flow rate for the dry sample.

When compared to the literature, a DSC study using low-denatured pea protein isolate and a heating rate of 5 °C/min from 20 to 110 °C obtained similar results [75]. The thermogram obtained in the literature has only one broad endothermic peak at around 78.5 °C [75]. Another DSC study using field peas at a pea starch-to-water ratio of 1:2 and a heating rate of 10 °C from 30–100 °C also obtained similar results of endothermic peaks from 75.5–89.9 °C [76]. Some of the literature reports both weaker and stronger peaks, possibly due to the denaturation of different components in peas, such as legumin and vicilin [77]. The multiple peaks are not present in the observed data, which may be due to the endothermic start-up hooks, which can diminish the detectability of weaker peaks [71]. Further, previous heat treatment on the sample source, such as spray-drying, may have had thermal effects on the sample, which would influence the shape of the DSC curve [77].

The pea protein and water DSC run produced an endothermic peak at around 89 °C. This value aligns with the high protein sample in the literature despite having a different pea-to-water ratio of 1:2 versus 1:1 [76]. The peak is more distinct than the dry peaks, which, again, suggests that increased water content results in an increase in denaturation enthalpy [70]. Unlike other samples, the paste curve appears to have a higher thermal stability than the powder curves, as the peak is at a higher temperature.

#### 3.3.4. Brown Rice Protein DSC Results

The rice protein samples were run under three different DSC conditions: dry rice protein powder using 30 mL/min N_2_ and 50 mL/min N_2_, and a 50–50 ratio of rice protein and deionized water using 50 mL/min N_2_. The rice protein (30) thermogram appears to calibrate at the beginning of the run and then have a consistently increasing slope. It is difficult to identify any meaningful temperature trends for this range. The rice protein (50) thermogram has a similar smooth and amorphous shape to the other dry samples but with a different slope. A stable, plateaued temperature range can be observed between 87–97 °C for the rice protein (50) curve. There may be a glass transition occurring around 100 °C as the slope of the curve decreases following this point [66]. A study using rice protein derived from long-grain rice combined with soy protein produced curves very similar to rice protein (50) [78]. In this study, a sample with a 1:0.1 ratio of rice protein-to-soy protein produced a very smooth and amorphous curve [78]. However, 2 mg of the sample was mixed with 10 μL of distilled water and allowed to reach full hydration [78]. Therefore, it may not be a completely accurate comparison; however, this finding in the literature does demonstrate the possibility of amorphous curves and highlights the significance of sample preparation and the source [78].

The paste curve reaches a steady baseline and has a more distinct endothermic peak at 65 °C. Again, the water content of the paste sample appears to increase the denaturation enthalpy [70]. A DSC study using three rice protein concentrates and a heating rate of 5 °C between 5 °C and 100 °C in an aluminum pan obtained peak temperatures of 63.6–70.2 °C [65]. These values align with the peak value of the paste; however, the literature states the samples were powders [65]. Regardless, obtaining a peak temperature within the range of the literature confirms that 65 °C is a reasonable endothermic peak temperature for rice protein.

### 3.4. Viscosity

**Figure 7:**
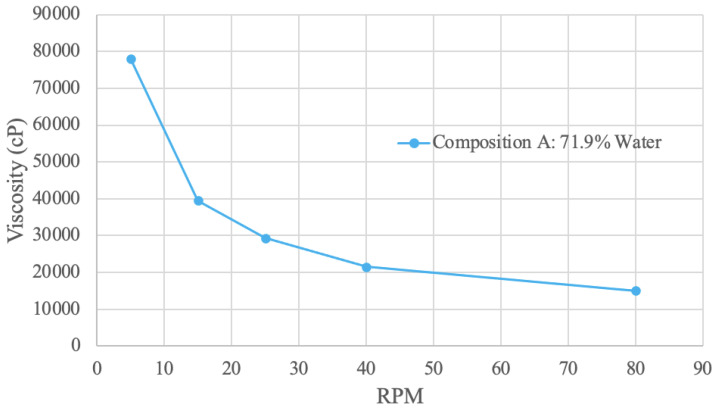
Spirulina Viscosity Curve.

**Figure 8:**
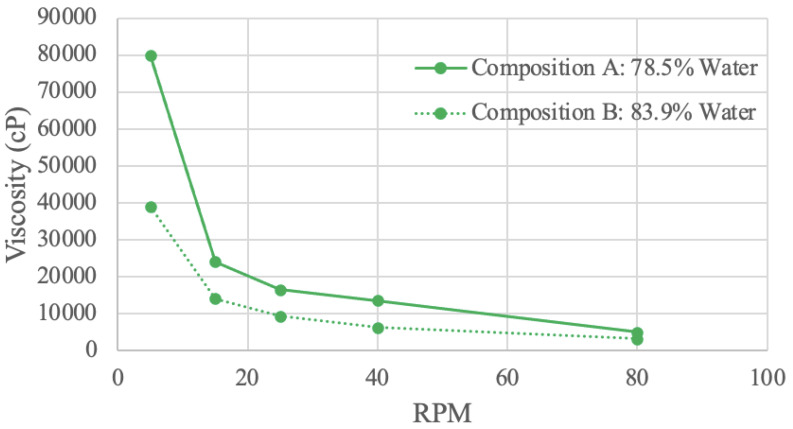
Soy Protein Viscosity Curve.

**Figure 9:**
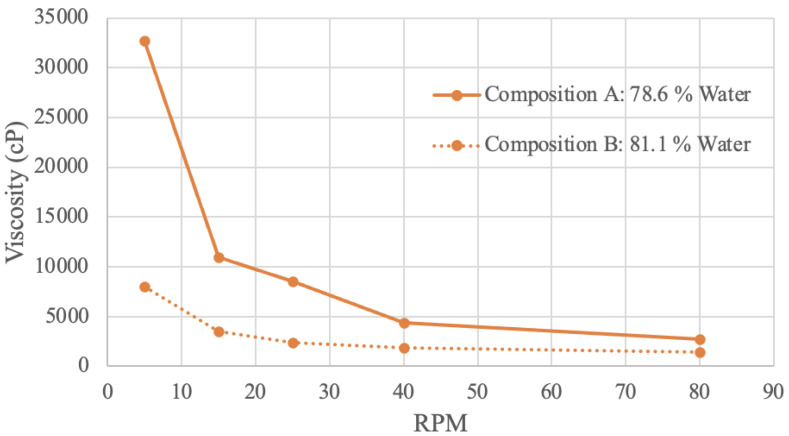
Pea Protein Viscosity Curve.

**Figure 10:**
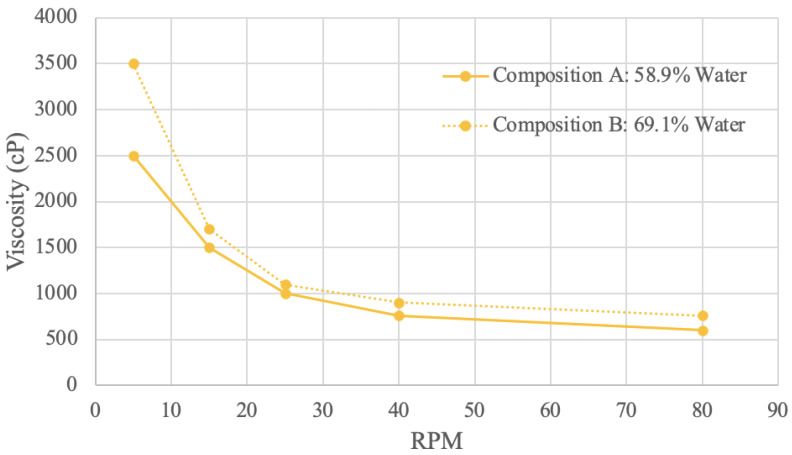
Brown Rice Protein Viscosity Curve.

Viscosity (in cP) at various RPM values was graphed (Figure 7, Figure 8, Figure 9 and Figure 10). Viscosity curves often compare viscosity (units of cP or mPa·S) to shear rate (units of s^−1^). For a concentric cylinder viscometer, the shear rate is equal to 1.7 times that of the RPM value of the outer cylinder [79]. For the purposes of this analysis, the shear rate is taken as being proportional to RPM; when RPM increases, so does the shear rate [79].

#### 3.4.1. Spirulina Viscosity Results

The spirulina sample viscosity results were obtained with a water concentration of 71.9% (Figure 7). The curve shape is exponentially decreasing; as RPM increases, viscosity decreases. Therefore, the sample exhibits shear-thinning behavior since the RPM is proportional to the shear rate [79]. At 5 RPM, the viscosity value is 78,000 cP, and this decreases to 15,100 at 80 RPM. The spirulina sample was also tested at 65% water; however, the viscosity was too high and could not be measured. This result is expected, as the increased solid concentration correlates with an increase in viscosity because of stronger intermolecular bonds [80]. Conversely, an increase in water—the solvent—decreases the viscosity of the solution.

Similar trend results were obtained in a study using a 2% spirulina concentration in a juice sample [81]. In this study, the viscosity versus shear rate curve had a decreasing slope, with viscosity values ranging from 3–5 mPa·s [81]. However, similar viscosity values were not obtained, which is attributed to the different solutions and lower spirulina concentration. Further, the addition of spirulina in the juice sample significantly increased the viscosity of the juice [81]. This observation from the literature aligns with the result of the 65% water sample having a greatly increased viscosity.

#### 3.4.2. Soy Protein Viscosity Results

The soy protein samples were tested in a viscometer using two different concentrations (Figure 8). Composition A has 78.5% water, and composition B has 83.9% water. At all RPM values, composition A is more viscous than composition B, most likely due to the increase in intermolecular forces and friction with a higher solute concentration [80].

At 5 RPM, compositions A and B have viscosities of 80,000 cP and 39,000 cP, respectively. These values continually decrease until 80 RPM, where composition A has a viscosity of 5000 cP and composition B has a viscosity of 3200 cP. The decreasing exponential curve shape is characteristic of certain pseudo-plastic polymer proteins, including soy protein isolate [82,83]. The downward trend indicates shear-thinning behavior since viscosity decreases with increasing RPM [82]. Further, composition A has a steeper slope than composition B. This observation suggests that concentration influences shear-thinning behavior, as a higher concentration has more entanglement [82].

A study investigating the response of viscosity to shear rate using various soy protein and sodium bisulfate (NaHSO_3_) solution concentrations also resulted in a decreasing exponential curve shape [84]. This study illustrated shear-thinning viscosity that decreased with an increase in the NaHSO_3_ solvent [84]. The viscosity of the soy protein-NaHSO_3_ solution was attributed to the various forces within soy protein, such as disulfide bonds, hydrophobic forces, and electrostatic interactions [84]. The influence of these forces on viscosity would decrease with a lower solid concentration, which was exhibited in the study and in the obtained results [84].

#### 3.4.3. Pea Protein Viscosity Results

Pea protein samples were tested in a viscometer with two different concentrations (Figure 9). Composition A has 78.6% water, and composition B has 81.1% water. At all RPM, composition A was found to be more viscous. This result again demonstrates that decreased water content tends to result in increased viscosity.

At an RPM of 5, composition A has a viscosity value of 32,700 cP, while composition B has a viscosity level of 8000 cP. At an RPM of 80, composition A’s viscosity decreases to a value of 2700, and composition B’s value decreases to 1400. This downward trend may be attributed to the protein particles expanding once they absorb water, resulting in a viscous flow, that then decreases with more rotations and mechanical energy [85]. As illustrated in the graph, when RPM increases and viscosity decreases, the sample is shear-thinning [79]. Further, composition B appears less affected by shear-thinning behavior, as the slope of the curve is overall less steep. This finding implies that shear-thinning behavior is proportionally related to concentration, likely due to molecular bonds and entanglement [82].

A study analyzing the RVA viscosity of pea protein slurry at 15% *w*/*w* produces a similar exponential curve shape [85]. The viscosity value begins at 2000 mPa·s, decreasing over time to 500 mPa·s, which is similar to the obtained results [85]. The differences in the initial viscosity value may be attributed to the preparation of the sample. In this study, the protein slurry was preheated to 50 °C and 95 °C [85]. Heating will influence viscosity, as viscosity is directly related to temperature [86]. This literature confirms that increased mechanical energy into the fluid, whether that be with increased time or RPM, is a factor that results in a less viscous fluid [85]. A different study investigating the relationship between shear rate and viscosity for pea protein isolate with 35% moisture content also found the sample to be shear-thinning [87].

#### 3.4.4. Brown Rice Protein Viscosity Results

Rice protein samples were tested in the viscometer using two different concentrations (Figure 10). Composition A had 58.9% water, and composition B had 69.1% water. The rice viscosity results are the only measured samples where a lower powder concentration had a higher measured viscosity. In other words, an increase in water concentration resulted in an increase in viscosity. This observation suggests that the rice protein sample may have different hydrogen bonding patterns, resulting in different velocity distortion compared to the other materials tested [80]. Other compounds within the commercial rice protein, such as carbohydrates, may also interfere with the bonding behavior. A study involving homogenized rice protein and starch samples at various concentrations also produced results where the addition of protein decreased viscosity [88]. Since the rice protein used to make compositions A and B was commercially obtained, it is not unreasonable to assume that possible previous treatments, such as homogenization, may have taken place, which would influence the viscosity.

Both viscosity curves demonstrate that viscosity decreases as RPM increases, which indicates shear-thinning behavior. The increased stress from the raised mechanical energy disorganizes the molecule arrangement, which decreases viscosity [89]. At 5 RPM, composition A has a viscosity of 2500 cP, and composition B has a viscosity of 3500 cP. At 80 RPM, composition A has a viscosity of 600 RPM, and composition B has a viscosity of 760 RPM. Of all the materials sampled, both rice compositions have the least difference in viscosity levels. Shear-thinning viscosity curves for rice protein were also reflected in the literature, even with different sample preparation techniques [88,89]. This shear-thinning flow behavior suggests that the rice protein acts as a pseudoplastic fluid [88].

### 3.5. Physical Properties and Nutrition

**Figure 11:**
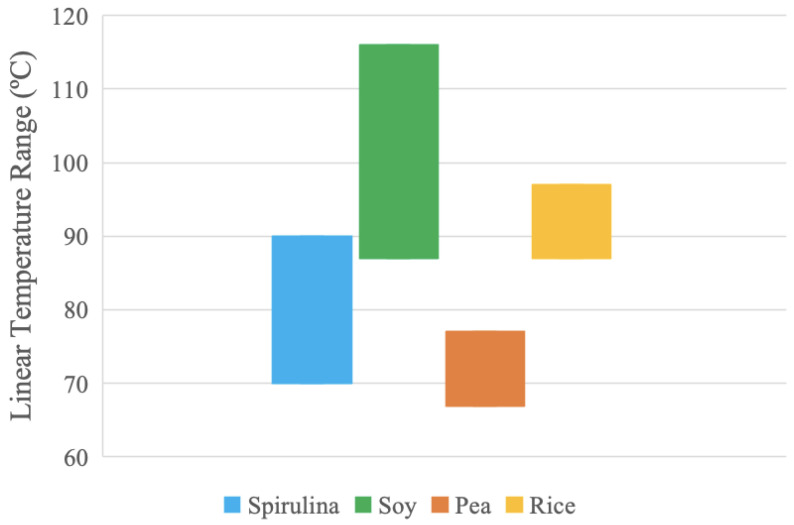
Linear temperature range for each protein sample.

**Figure 12:**
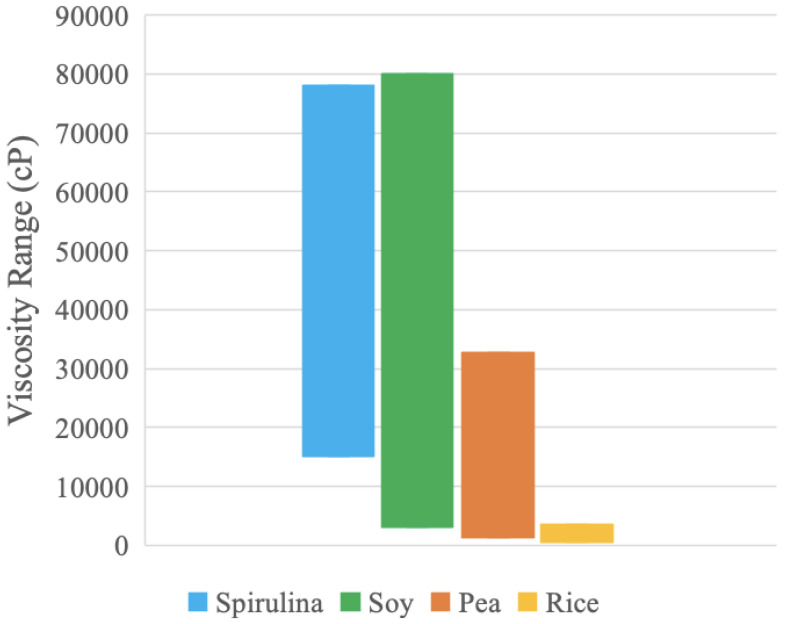
Viscosity range for each protein sample.

In order to summarize the material properties, Table 5 compares the alternative proteins to each other in terms of their linear processing temperature range and their viscosities. In addition, the Alibaba wholesale retail purchase cost is provided [39,45,50,55]. These ranges are graphed in Figure 11 and Figure 12.

The temperature ranges in Figure 11 were extracted from the linear trend present in the pure protein thermograms produced by the DSC analysis. These temperature ranges are assumed to be relatively more stable due to the lack of thermal activity. Optimum processing temperatures may be found in these regions as the protein samples have more predictable thermal behavior. The soy and spirulina samples have the largest stable temperature ranges. The results suggest that the soy sample can be processed at higher temperatures compared to the other samples due to increased thermal stability over a broader temperature range.

Figure 12 displays the viscosity ranges exhibited by both compositions of the protein samples between 5–80 RPM. Soy and spirulina exhibit the largest viscosity values and ranges and may be appropriate materials to use for processing using screw extruders, as these extruders are used for highly viscous non-Newtonian materials [90]. Rice has a much smaller range, which suggests that, of the samples, the viscosity of rice is the least influenced by water concentration. Rice may have different hydrogen bonding patterns than the other samples, which could result in a narrower range [80].

In both the temperature and viscosity data, soy and spirulina have the largest ranges. These protein samples can likely be used across more processing options and parameters compared to pea and rice. Moreover, the pea range is in the region of the spirulina range for both temperature and viscosity, which suggests that pea has similar thermal and rheological responses to spirulina and can possibly be used as a less-expensive substitute. These materials can also be mixed to provide composite protein sources with various beneficial properties (e.g., amino acid profiles, although it should be noted that there are many complexities with amino acids, such as bio-availability, including digestibility, availability, and absorption, which is left for future work).

Various plant protein options contain multiple essential amino acids, and content varies greatly across plant protein sources. Spirulina contains essential amino acids such as leucine and tryptophane; the superfood also has non-essential amino acid content, including glutamic acid and aspartic [91]. Minerals like potassium, calcium, phosphorous, magnesium, zinc, and iron are present in spirulina in significant concentrations [91]. Spirulina also contains vitamin B12, which is notable since animal products tend to be the greatest source of B12 [91]. Despite the richness in vitamins and minerals and the presence of multiple amino acids, microalgae fall short of the recommended essential amino acid percentage at 23% [92]. Therefore, if spirulina (algae protein) was the only protein source consumed, the essential amino acid requirement would not be met.

Legumes, such as soybeans and peas, are generally good sources of protein, carbohydrates, fiber, vitamins, and minerals. Of the essential amino acids, histidine, isoleucine, leucine, lysine, threonine, and valine are generally present in legumes [93]. Certain amino acids—methionine, phenylamine, and tryptophan—are not as abundant across legumes [94,95]. Gorissen et al. demonstrated that soy and pea amino acid percentage levels of 27% and 30%, respectively, meet the WHO/FAO/UN amino acid percentage level, based on protein consumption of 0.66 g/kg body weight/day [92]. These levels, however, are lower than the overall animal-based protein essential amino acid percentage (37 ± 2%) [92].

Cereal grains, such as rice, are widely consumed and are a readily available protein source globally. Essential and non-essential amino acids are present in certain strains of rice, such as rice bran and brown rice [96,97]. Generally, the limiting amino acids in cereals are lysine, methionine, and threonine [98]. Brown rice also meets the WHO/FAO/UNU amino acid requirement, with an essential amino acid percentage in total protein of 28% [92]. This value is also lower than the animal-based protein percentage [92].

## 4. Discussion

### 4.1. Plant Protein Processing Options

Heating is a widely used food processing technique, especially in cooking, and it has demonstrated positive impacts on plant protein digestibility and nutritional quality [93,99]. Heating processes utilize thermal treatments for sterilization, flavor and texture enhancement, improvements in functional properties, such as emulsification, and the destruction of undesired compounds [93,100]. The adverse effects of thermal treatment have also been observed, such as the degradation of protein and micronutrients, which can alter amino acid composition [93]. However, protein digestibility can be improved through the denaturation of proteins by heating [93]. The temperature processing parameters for cooking with plant protein usually occur around 100 °C [93,100,101]. Therefore, cooking may be a feasible processing method for compounds comprised of soy and rice protein, as their linear temperature ranges were found to be 87–116 °C and 87–97 °C.

Drying is another common food processing method. The techniques include spray drying, freeze drying, and the use of supercritical fluid technology [102]. In terms of protein functional properties, drying technology is commonly used to improve emulsifying and foaming properties, protein solubility, and water-holding capacity [93]. Spray drying inlet temperatures are typically well over 100 °C; therefore, each protein sample in this study may experience thermal effects if used in a drying process [103,104]. Vacuum drying, however, can also be effective below 100 °C [105].

Autoclaving can be used as a sterilization technique and is a high-pressure cooking method [93]. This process utilizes steam, which is important to note, as thermal stability can be affected by water concentration [70]. The typical autoclaving process parameters for plant-based proteins are 5–15 psi, 112–127 °C, and 10–50 min [93,99]. Therefore, the soy protein sample may be a possible candidate for autoclaving processing.

Extrusion combines mechanical shear, pressure, and heat by using a large rotating screw and high temperatures to result in a high-temperature short time (HTST) food processing technique [106]. Materials extrusion is also a method that can be coupled with additive manufacturing. The denaturation, unfolding, and realignment of plant protein molecules can be present in extrusion, which can improve the functionality of the compound and produce a meat-like texture [106]. The high temperature in extrusion leads to hydrogen and intramolecular disulfide bond breakage, which can promote the formation of new protein aggregates [107]. Overall, extrusion temperatures have a large range but typically fall in the 90–200 °C span, and the screw speeds have RPM values in the hundreds [106]. Zahari et al. produced a meat analogs product by using various soy protein isolate and hemp protein concentrate mixtures at extrusion temperatures ranging from 40–100 °C and screw speeds of 300–800 RPM [108]. Due to the encompassing temperature range, extrusion is a feasible processing method for the spirulina, soy protein, pea protein, and rice protein samples used in this study. The increased industrial RPM values, however, would have mechanical effects by decreasing the viscosity of the protein isolates. Moreover, particle size has also been demonstrated to influence extrusion. Carvalho et al. found that corn meal extrudate expansion increased with particle size, and that the mechanical resistance of the extrudates was significantly larger for the smallest particle size than the largest particle size [109]. If this same result is extended to all plant proteins, the rice protein sample would have the highest level of expansion, and the soy protein sample would have the most mechanical resistance based on the results of the materials used in this study. Moreover, increased density correlates with decreased viscosity, which would influence extrusion behavior [110].

### 4.2. Viability and Sustainability

Compared to an ovo-lacto vegetarian diet, the average meat-based American diet requires more land, energy, and water resources [5]. Vegetarian diets have been demonstrated to produce less per capita GHG emissions than Mediterranean and pescetarian diets and the average global diet in 2009 [111]. Further, a vegetarian diet is likely to require less additional cropland than meat-including diets [111]. A life cycle analysis performed by Detzal et al. found that a meat-analogous plant-based extrudate had a lower carbon footprint in terms of carbon dioxide equivalents than chicken when using a functional unit of 30 g of protein and 30 g of product [17]. In the same study, when compared to chicken, an optimized plant-based meat alternative had lower impacts in environmental categories, including water processing, land use, climate change, eutrophication, acidification, particulate matter, ozone depletion, and oxidants formation, and was comparable in cumulative nonrenewable energy demand [17]. Additionally, crops, such as soybeans, are used to feed livestock and poultry [112]. Of all soya and grain produced, 75% and 40% is directed towards animal feed, respectively [112]. Countries with a high level of animal products consumed, especially beef, have a larger area of agricultural land usage [113]. Alexander et al. found that the global consumption of an average American omnivorous diet would require 178% more land for agriculture than is currently in use, highlighting the importance of plant-based food options that can be produced on a global scale [113].

The proteins chosen in this study—spirulina, soy, pea, and brown rice—are strong candidates for plant-based food and meat-analogous products because of protein content and geographic availability. Per Table 1, the average protein content as a percentage for spirulina isolate, soy protein isolate, pea protein isolate, and brown rice protein isolate is 67%, 88%, 78%, and 81%, respectively. Those values are higher than the reported plant protein contents for hemp at 51%, lupin at 61%, oat at 64%, and corn at 65% [92]. Further, the protein samples used in this study have a higher protein content than egg at 51%, and a comparable percentage to animal-based proteins of whey at 72–84%, milk around 75%, and casein at 67–78% [92]. Spirulina is found across the globe as it is naturally occurring in salt water, fresh water, brackish water, soil, sand, and marshes [114]. Soya beans are grown primarily in the Americas and Asia, with the top producers being the USA, Brazil, Argentina, China, and India [115]. Soya production is also scattered throughout Europe and Africa [115]. Green peas are mainly produced in Asia, with China and India dominating the production market; Europe, the Americas, and Africa also produce green peas [115]. Asia has the largest share of rice production, with China, India, and Southeast Asian countries as the top producers [115]. The Americas and Africa also have a smaller share of rice production [115].

### 4.3. Future Work

The literature clearly shows that there would be environmental and health benefits to reducing animal proteins in favor of plant and nonmeat-based proteins. Although nonmeat sources are less expensive per unit protein than animals, as was shown in the introduction, plant-protein based foods are generally more expensive at the retail level than animal proteins. This can be ascribed to a number of reasons, including processing costs. The results presented here are the first step in making nonmeat-based protein affordable at the retail scale, as the base thermal and rheological properties have been elucidated that can allow for greater competition between methods and providers, and lower costs can be assumed. Future work is needed to determine if these are valid assumptions. Further work and process testing, however, is needed to establish these protein isolates in a range of foods. For example, as the results here and elsewhere have conveyed that temperature and water percentage impact the viscosities of the materials, an in-depth study is needed on both the pure materials and protein mass percent combinations for the available temperature processing ranges and viscosity ranges summarized in Section 3.5. Future work could also assess the impacts of mixing the various volume fractions of the proteins to create ideal properties for a specific material-processing technique. In addition, the geographical availability of each protein source could be evaluated, both for the local optimization of protein source availability during agricultural times as well as considering their use as resilient food [116,117,118,119] to help provide adequate nutrition during emergencies [120]. Even excluding emergencies with rapid growth observed in the global population, the demand for meat is increasing and can be offset with plant-based meat alternatives that solve the economic, environmental, and health problems caused by the over-consumption of meat products by humans [121,122]. Far more work is needed to build upon the preliminary results here to meet the complex challenges of replacing meat entirely [122].

The results, however, can be compared to meat. Here, this will be carried out for chicken, as, in 2021, over 132 million tons of poultry meat was consumed globally, which makes it the most common meat-based food [123]. Normally, meat is not converted to a powder and consumed, so only the thermal and rheometric properties will be compared. First, consider that chicken meat in the form of chicken breast patties yields three endothermic transitions, with peak transition temperatures of 53 °C, 70 °C, and 79 °C, respectively [124]. If the alternative proteins analyzed here were to be processed with chicken meat in all cases, the linear temperature range (as shown in Figure 11) would result in endothermic transitions in chicken meat caused by the denaturation of myofibrillar at 53 °C [124]. Only pea protein could be further processed in its linear temperature range before the denaturation of chicken sarcoplasmic (70 and 79 °C) proteins, respectively [124]. Similarly, for the rheometric analysis, chicken meat is normally eaten simply cooked, although there have been some experiments for converting it to space food as a paste. When scientists investigated Chinese yam/chicken semi-liquid paste, they were able to obtain a wide range of viscosities ranging from honey to thick pudding by adding various proportions of chitosan [125]. This same approach could be used with the alternative proteins investigated here so that they could be used for more technically sophisticated processing techniques (e.g., 3D printing [126]). In addition, chitosan can have other beneficial properties, such as being used as a food preservative [127]. This property could also be explored, as it could lead to potentially new products (e.g., alternative protein pastes targeted for the growing 3D printing food market [128,129]). Combining these approaches is already underway, as demonstrated by Wang et al., who combined chicken paste and pea protein to make 3D-printed nuggets [130]. It is clear that the results from this study can also be used as a baseline for food 3D printing studies in the future. In addition, substantial work is underway to increase the aqueous solubility of plant proteins to provide alternative proteins [131]. Future work is needed to determine the impact on the viscosity and DISC of these and other functional properties of plant proteins. Finally, it should be stressed that when comparing alternative proteins to meat, it is the final processed (including cooking) material properties that are the most important for consumption; this area is also needed in the future.

## 5. Conclusions

This study extended the current state of research into alternative proteins by providing new base material properties for four globally common sources of alternative proteins. These material properties can be used to select a wider range of food processing techniques. Density and particle size were found for (a) spirulina, (b) soy protein, (c) pea protein, and (d) brown rice protein. These properties provide lower limits for resolution for the material-extrusion-based additive manufacturing of the materials. The spirulina sample had a density of 0.49 g/cm^3^, a mean area of 28 µm^2^, and a mean diameter of 6.0 µm. The soy protein sample had a density of 0.68 g/cm^3^, a mean area of 3.8 µm^2^, and a mean diameter of 2.2 µm. The pea protein sample had a density of 0.76 g/cm^3^, a mean area of 6.6 µm^2^, and a mean diameter of 2.9 µm. The brown rice protein sample had a density of 0.54 g/cm^2^, a mean area of 340 µm^2^, and a mean diameter of 21 µm. The DSC analysis provided thermal processing windows for the materials. The DSC analysis produced dry curves with an amorphous shape and paste curves with a more distinct endothermic peak. Linear temperature ranges were extracted and interpreted as processing parameters for food production. The linear temperature ranges were 70–90 °C for spirulina, 87–116 °C for soy protein, 67–77 °C for pea protein, and 87–97 °C for brown rice protein. Soy had the largest linear temperature range and is likely the most thermally stable due to fewer deviations. The viscosity analysis determined each protein sample experienced shear-thinning and that viscosity increased with decreased water concentration, with rice being an exception to the latter trend. The obtained viscosity range for spirulina was 15,100–78,000 cP, 3200–80,000 cP for soy protein, 1400–32,700 cP for pea protein, and 600–3500 cP for brown rice protein. Additionally, extrusion seems to be a viable method for the further processing of the protein isolates, as this technique has a large temperature operating range and variable screw speed. Extrusion has been used to develop meat-analogous products with plant proteins. Plant-based protein products can be used as an alternative to traditional poultry, livestock, and other meat proteins, as they have a large range of essential and non-essential amino acids and are considered more resource-efficient. The protein isolates analyzed in this study have high protein concentrations and wide geographic availability, making them strong candidates for further research into the materials processing and economic viability of extruded meat-analogous products. Future work will include process optimization and extending this to mixtures of alternative protein materials.

## Figures and Tables

**Table 1:** Protein cost (per gram), ranging from bulk costs to retail purchase costs.

Protein	Protein/Mass g/kg	Purchase Cost USD/kg	Protein Cost USD/kg	Sources
Boneless/Skinless Chicken Breast	310	6.28	0.0203	[31,32]
Beef Patties	230	13.69	0.0595	[33,34]
Pasture Raised Pork Shoulder	231	19.93	0.0863	[35,36]
Ground Lamb	166	15.71	0.0946	[37,38]
Alibaba Wholesale Spirulina Isolate Powder	600	8.50	0.0142	[39]
Spirulina Powder	667	95.29	0.143	[40,41]
Retail Spirulina Powder	667 ^1^	99.98	0.150	[40,42]
Soybeans	433	0.52	0.00120	[43,44]
Alibaba Wholesale Soy Protein Isolate Powder	900	3.25	0.00361	[45]
Bulk Food Store Wholesale Soy Protein Isolate Powder	900	20.68	0.0230	[46]
Retail Soy Protein Isolate Powder	833	25.66	0.0308	[47]
Green Pea	54.2	0.31	0.00572	[48,49]
Alibaba Wholesale Pea Protein Isolate Powder	800	3.00	0.00375	[50]
Bulk Food Store Wholesale Pea Protein Isolate Powder	800	20.68	0.0259	[51]
Retail Pea Protein Isolate Powder	727	45.19	0.0622	[52]
Crude Rice Bran	134	0.18	0.00134	[53,54]
Alibaba Wholesale Brown Rice Protein Isolate Powder	850	2.50	0.00294	[55]
Bulk Food Store Wholesale Brown Rice Protein Isolate Powder	785	17.34	0.0221	[56]
Retail Brown Rice Protein Isolate Powder	809	43.21	0.0534	[57]

^1^ Protein concentration not listed on product site, taken from USDA.

**Table 2:** Viscometer sample water concentrations.

Protein	Composition A (% Water)	Composition B (% Water)
Spirulina	65.5	71.9
Soy	78.5	83.9
Pea	78.6	81.1
Brown Rice	69.1	58.9

**Table 3:** Dry density of alternative proteins.

Protein	Density (g/cm^3^)
Spirulina	0.49
Soy	0.68
Pea	0.76
Brown Rice	0.54

**Table 4:** Particle mean area, the mean diameter, and the literature diameter values for the protein powders.

Protein	Mean Area (µm^2^)	Mean Diameter (µm)	Literature Diameter Values (µm)
Spirulina	28	6.0	<125 [62]
Soy	3.8	2.2	0.1–100 [63]
Pea	6.6	2.9	1.7–270 [64]
Brown Rice	340	21	23–150 [65]

**Table 5:** Summary of linear temperature and viscosity ranges.

Protein	100% Powder Sample Linear Temperature Range (°C)	Viscosity Range (cP)	Wholesale Retail Purchase Cost (USD/g protein) (Table 1)
Spirulina	70–90	15,100–78,000	0.0142
Soy	87–116	3200–80,000	0.00361
Pea	67–77	1400–32,700	0.00375
Brown Rice	87–97	600–3500	0.00294

## Data Availability

The original contributions presented in the study are included in the article, further inquiries can be directed to the corresponding author.
